# Simultaneous occurrence of follicular and papillary thyroid carcinomas in same thyroid lobe: A case series of six patients from Qatar

**DOI:** 10.1016/j.ijscr.2020.06.070

**Published:** 2020-06-20

**Authors:** Abdelrahman Abdelaal, Walid El Ansari, Abdelrahman Abusabeib, Hanan Farghaly, Abdelhakem A.M. Tabeb

**Affiliations:** a Department of General Surgery, Hamad General Hospital, Hamad Medical Corporation, Doha, Qatar; b Department of Surgery, Hamad General Hospital, Hamad Medical Corporation, Doha, Qatar; c College of Medicine, Qatar University, Doha, Qatar; d School of Health and Education, University of Skövde, Skövde, Sweden; e Department of Lab Medicine & Pathology, Hamad General Hospital, Doha, Qatar

**Keywords:** Follicular thyroid carcinoma, Papillary thyroid carcinoma, Differentiated

## Abstract

**Background:**

Papillary thyroid carcinoma (PTC) and follicular thyroid carcinoma (FTC) are the first and second most common thyroid cancers comprising about 85% and 10% of all thyroid cancers. Simultaneous occurrence of medullary and papillary thyroid cancer has been reported with various presentations, but simultaneous occurrence of FTC in addition to PTC as differentiated cancers, is an unusual event that is rarely reported.

**Presentation of cases:**

We report our experience of six rare cases of synchronous coexistence of FTC and PTC with unique features. Case 1 is 31 old Egyptian female. Case 2 is a 61 year old Sudanese male. Case 3 is a 59 year old Sudanese male. Case 4 is a 56 years old Indian female. Case 5 is a 35 years old Filipina female. Case 6 is a 52 years old Qatari female. The six cases are special in their co-occurrence of two thyroid carcinoma, consisting of histologic features of follicular thyroid carcinomas, and classical papillary thyroid carcinoma, possibly the first case series of simultaneous occurrence of these two types of thyroid cancer in the Middle East and North Africa Region.

**Conclusions:**

We present rare cases of concurrent FTC and PTC. These six cases add more data highlighting the coincidental simultaneous coexistence of FTC and PTC. Endocrinologists and pathologists should be aware of and vigilant to this variety.

## Highlights

• Simultaneous occurrence of papillary and follicular thyroid carcinomas in the same lobe are very rare.
• First case series of simultaneous occurrence of these two types of thyroid cancer in the Middle East and North Africa Region.
• Endocrinologists and pathologists should be aware of and vigilant to this variety.
• Occurrence of both types together shifts patients from low risk to intermediate or high risk which may reflect on management.

## 1 Background

Although composite thyroid carcinomas have been reported in the literature, the simultaneous occurrence of multiple thyroid tumors of different histopathological types in the same thyroid lobe is a rare presentation and known as mixed, hybrid tumours or composite tumours [1].

About 71 cases of concurrent papillary thyroid cancer (PTC) and medullary thyroid cancer (MTC) have been reported [2], but cases of PTC and follicular thyroid cancer (FTC) presenting synchronously are much rarer [[3], [4], [5]] and signify the simultaneous occurrence of distinctly different entities. Well-differentiated thyroid carcinomas (e.g., PTC and FTC) are usually sporadic in most cases [6], and the coexistence of two independent and simultaneous follicular epithelial cell carcinomas, a papillary carcinoma and a follicular carcinoma, is extremely rare [7].

To the best of our knowledge this could be the first case series of simultaneous occurrence of two types of thyroid cancer (PTC and FTC) reported from the Middle East and North Africa region (MENA). One case report from the Middle East region had three types of thyroid cancer [6]. We report these cases due to the uniqueness of their histopathological findings and to debate their possible complex histogenesis. This case series report is in line with the updated consensus-based case series (PROCESS) guidelines [8].

## 2 Case presentations

### 2.1 Case 1

**Fig. 1:**
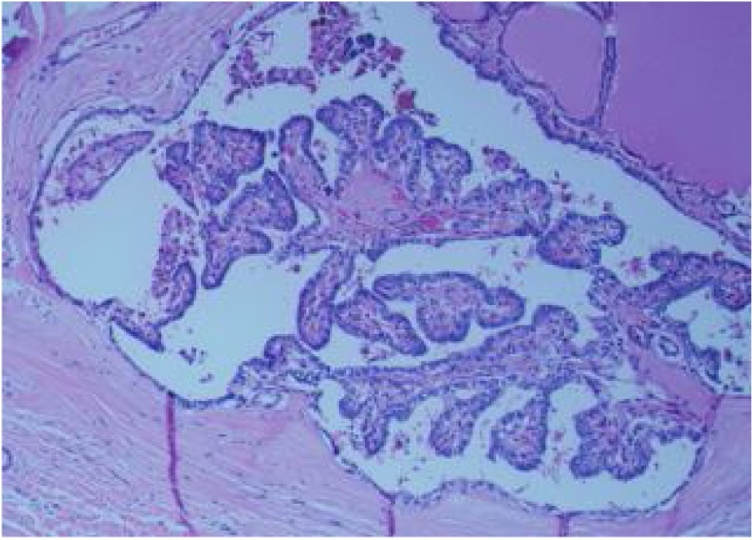
Papillary thyroid carcinoma with characteristic nuclear features (nuclear crowding, overlapping, clearing, membrane irregularities and inclusions).

**Fig. 2:**
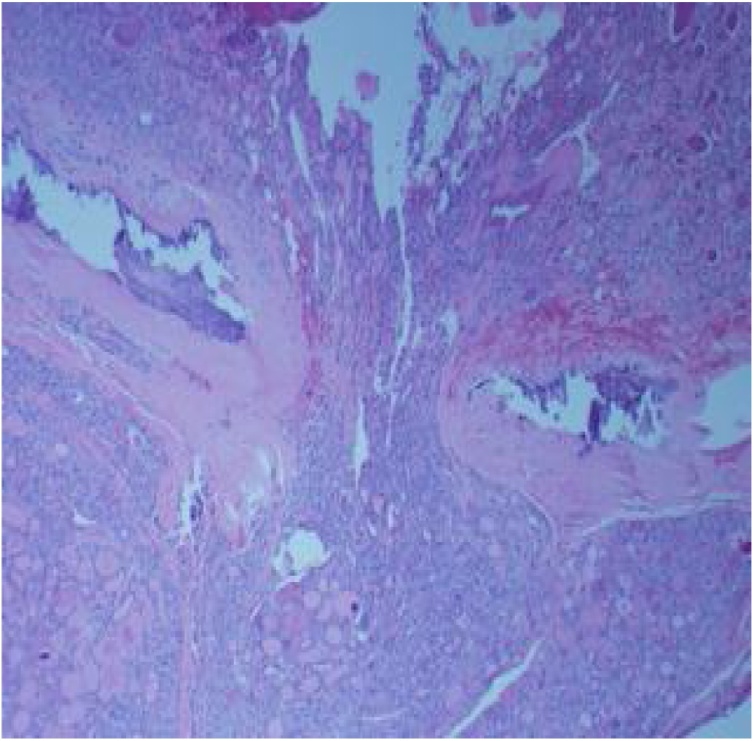
Thyroid follicular carcinoma widely invading the thyroid capsule.

An Egyptian female, 31 years old, presented to our thyroid clinic at Hamad Medical Corporation (biggest tertiary care facility) in Doha, Qatar, with left neck swelling since a year, increasing in size, associated with mild left neck pain. She had no history of irradiation therapy and no family history of cancer thyroid. Examination revealed a left neck thyroid nodule (4 × 3 cm) that moved with swallowing, and no palpable lymph nodes. Investigations showed normal thyroid function tests (TFTs). Ultrasound (US) of the thyroid revealed a large left-lobe thyroid nodule (5 × 2.5 cm) with small thin peripheral halo, peripheral and central vascularity and coarse calcifications. Ultrasound guided fine needle aspiration (FNA) showed follicular cells of undetermined significance (FLUS). The patient underwent left hemithyroidectomy. Post-operative histopathology showed left papillary thyroid carcinoma (PTC) (5 × 4 cm) (Fig. 1) and follicular thyroid carcinoma (FTC) (1.3 cm) (Fig. 2). The FTC had uninvolved margins, the tumor was very close to the posterior and anterior margins (within 0.1 mm), and AJCC staging [9] was pT3, N0. The PTC histology was oncocytic, with G1, well-differentiated histologic grade, was adjacent to the anterior margin, and AJCC pathologic staging [9] was pT1b, N0. Hence, the patient underwent completion right hemithyroidectomy, and histopathology revealed benign thyroid with chronic lymphocytic thyroiditis. She then received two fractionated doses of radioactive Iodine (30 mci). Follow up radioactive whole body scan showed no evidence of radioiodine avid local or distant pathology, and follow up US of the neck showed no definite residual or recurrence in the thyroid bed. Laboratory results after two years showed very low thyroglobulin (<0.1 ng/mL) and thyroglobulin antibodies (<0.9 IU/mL).

### 2.2 Case 2

A Sudanese male, 61 year old, was being followed up at the urology department for a recent radical prostatectomy for prostatic cancer that was followed by radiation therapy. During follow up, CT scan of the chest showed an incidental finding of an enlarged right thyroid lobe that had a central area of hypodensity. The patient was referred to our thyroid clinic. On examination, we found a right thyroid mass. There was no family history of cancer thyroid. Investigations showed normal TFTs. Ultrasound of the thyroid gland showed enlarged right thyroid lobe (3.1 cm anterio-posterior dimension) that contained a large, mainly isoechoic, heterogenous nodule occupying almost all of the right thyroid lobe, with slightly increased peripheral vascularity. The left thyroid lobe measured 1.5 cm (anterio-posterior dimension), showed normal echotexture and normal flow on colour Doppler, and contained multiple small nodules, the largest of which appeared cystic in the lower pole measuring 1 × 0.7 cm. Ultrasound guided FNA of the right thyroid raised suspicion for follicular neoplasm. The patient underwent right hemithyroidctomy. Histopathology indicated unifocal FTC (6 × 3 × 2.7 cm), encapsulated, uninvolved margins, angio-invasive (focal < 4 vessels), no lymphatic or perineural invasion and no extrathyroidal extension. Pathologic stage (pTNM, AJCC eighth edition) [9] was pT3a Nx. The specimen was also sent for review at the Mayo Clinic which indicated a well-differentiated follicular neoplasm with angioinvasion and capsular invasion, most consistent with follicular carcinoma, with a separate focus of papillary thyroid microcarcinoma (3 mm), pathologic stage pT1a Nx [9]. The patient underwent completion left hemithyroidectomy. Histopathology indicated nodular hyperplasia with predominant nodule on a background of chronic non-specific thyroiditis. The patient received high dose (100 mci) radioactive Iodine ablation (RAI). Follow up whole body scan 3 months later showed no evidence of either residual thyroid tissue or metastatic tumor; and follow up US pf the neck 1 year later showed no thyroid tissue residual, or focal lesion at the thyroid bed. Laboratory results after 15 months showed very low thyroglobulin (<0.2 ng/mL) and thyroglobulin antibodies (<0.9 IU/mL).

### 2.3 Case 3

A Sudanese male, 59 year old, presented to our thyroid clinic with recurrent multinodular goiter involving mainly the isthmus and left lobe. He had history of thyroid surgery 20 years back in Sudan, no history of irradiation therapy and no family history of cancer thyroid. On examination, the patient was clinically euthyroid, with a huge frontal neck swelling (15 × 7 cm). Investigations showed normal TFTs. US of the thyroid showed absence of right thyroid lobe, and the left lobe and isthmus were enlarged with multiple nodules, showing solid complex echotexture with partially ill-defined margins and central areas of anechoic components suggestive of cystic degeneration, the largest of which measured 4.1 × 2.8 cm. Ultrasound guided FNA showed follicular lesion of FLUS on a background of lymphocytic thyroiditis. Completion left thyroidectomy was done. Histopathology showed FTC, widely invasive (5 cm), abutting the inked anterior margin and 0.1 mm from inked posterior resection margins, with lymphovascular invasion but no perineural or angioinvasion, no extrathyroidal extension, of stage pT3a pNx [9]. There was also a separate focus of PTC (greatest dimension = 1.5 cm) of stage pT1b pNx [9], abutting the inked anterior margin, with no lymphovascular, perineural or angioinvasion, no extrathyroidal extension. The patient was discussed at our thyroid multi-disciplinary meeting (MDT) and was categorized as high risk stratification (ATA 2015). He received RAI 100 mci, then follow up US showed residual thyroid tissue, so the patient received another 30mci RAI. Follow-up US of the neck after 22 months showed no residual thyroid tissue, and both thyroid beds were normal. Final laboratory findings showed thyroglobulin 3.6 ng/mL, and thyroglobulin antibodies 1.2 IU/mL.

### 2.4 Case 4

An Indian female, 56 year old, with Hodgkin's lymphoma in remission since 2001. Presenting at our thyroid clinic, she had noticed a left side neck swelling since one year and started feeling pressure symptoms since one month. Examination revealed a bilateral neck swelling that moved with swallowing. She had no history of irradiation therapy and no family history of thyroid cancer. Investigations showed normal TFTs. Follow up whole body fluorodeoxyglucose positron emission tomography integrated with computed tomography (FDG PET CT) showed no signs of lymphoma relapse or lymph node or distant organ metastasis, but showed incidental highly FDG positive bilateral thyroid nodules. US of the thyroid showed left thyroid nodule (4.5 cm) with a smaller nodule within it with heavy rim calcification and solid component with microcalcification at the bottom. US of the neck also showed 2 hypoechoic nodules in the right lobe, the largest was ill-defined with coarse calcification measuring (7 × 7 × 10 mm). No suspicious lymph nodes were seen. Ultrasound guided FNA showed atypical follicular lesion of undetermined significance (AUS). The patient underwent total thyroidectomy, and histopathology showed FTC, minimally invasive and multifocal classical variant PTC. The FTC in the left lobe was unifocal (4.5 × 3.5 × 2.5 cm), minimally invasive, < 0.1 mm from the posterior margin, no perineural invasion or angioinvasion, but present lymphatic invasion, and no extrathyroidal extension. Pathologic stage was pT3a pNx [9]. The PTC was multifocal with nodular hyperplasia, present in both lobes (first focus in right lobe 1 × 0.8 × 0.7 cm, 0.1 mm from the anterior margin; second focus in right lobe 0.5 cm in maximum dimension; third focus in left lobe 0.6 cm in maximum dimension). No lymphatic, perineural or angioinvasion or extrathyroidal extension. Pathologic stage was mp T1a pNx [9]. The patient was discussed in our thyroid MDT meeting and categorized as high risk stratification (ATA 2015) eligible for high dose radioactive iodine ablation. The patient travelled out of the country and was lost to follow up.

### 2.5 Case 5

A Filipina female, 35 years old, was referred to our thyroid clinic with multinodular goiter and a dominant nodule in the right lobe. She complained of mild hoarseness of voice, but no pressure symptoms. There was no family history of thyroid cancer and no history or irradiation. On examination, there was right thyroid lobe enlargement. She was clinically euthyroid, with normal TFTs. US of the neck showed heterogeneous echopattern with mild increased vascularity and multiple nodules with calcification in both lobes. There were at least three nodules in the right lobe, the largest complex nodule was in the lower pole (2.1 × 1.9 cm), and another solid nodule with calcification in the upper pole (1.8 × 1 cm). The left lobe also showed multiple nodules, where the largest complex nodule measured 4.8 × 2.5 cm. There were no significantly enlarged cervical lymph nodes. FNA (ultrasound guided) showed right thyroid nodules comprising malignant cells consistent with PTC. The patient underwent total thyroidectomy. Histopathology showed right micro PTC, multifocal (two foci), largest was at least 0.8 cm, with uninvolved margins, no lymphovascular, perineural, or angioinvasion and no extrathyroidal extension, and pathologic staging was pT1aN0 [9]. Histopathology also showed right FTC, unifocal, at least 1.3 cm, with uninvolved margins, no lymphovascular, perineural or angioinvasion and no extrathyroidal extension, and pathologic staging pT1bN0 [9]. The patient was discussed at our thyroid MDT meeting and categorized as high risk stratification (ATA 2015) eligible for high dose radioactive iodine ablation. The patient travelled out of the country and was lost to follow up.

### 2.6 Case 6

A Qatari female, 52 years old, presenting with a neck swelling a year ago, associated with some pain on swallowing, no compression symptoms, underwent an FNA in Thailand that showed suspicious follicular lesion and was hence refered to our thyroid clinic. On examination, there was a nodular swelling of the thyroid gland, but it was not tender. US of the thyroid showed multiple right lobe solid thyroid nodules, largest was complex, predominantly solid (15 × 26 mm), showing rim calcification, cystic change, and intra nodular vascularity. The left lobe measured 21 mm, and had an isoechoic thyroid nodule, (17 × 20 mm) showing hypoechoic halo and intra nodular vascularity. There were a few cervical lymph nodes with preserved echogenic hilum. Ultrasound guided FNA was repeated at our institution, the right thyroid nodule was FLUS, and the left thyroid nodule was also FLUS. The patient was discussed at our thyroid MDT and planned for total thyroidectomy which was undertaken. Histopathology revealed right lobe single focus FTC (2.7 × 1.5 × 1.5 cm) with capsular invasion, margins were free of carcinoma, < 0.5 mm from both anterior and posterior margins, no lymphovascular, perineural, or angioinvasion no extrathyroidal extension, and pathologic staging was pT2NX [9]. There was also a single focus papillary thyroid microcarcinoma, follicular variant (0.8 × 0.6 × 0.4 cm), non-capsulated, margins were uninvolved by the carcinoma, closest margins were the anterior and posterior margins with < 0.5 mm clearance, no lymphovascular, perineural, or angioinvasion, no extrathyroidal extension, and pathologic staging was pT1aNX [9]. In addition, the histopathology showed an adenomatoid nodule in the left lobe (third lesion), classified as pT1a [9]. The patient was discussed at our thyroid MDT meeting, and she was straified as intermediate risk (ATA guideline). She was planned for low dose RAI ablation and thyroid cancer surveillance with neck US and thyroglobulin tumor markers, and suppressive therapy with Levothyroxine. US of the neck post total thyroidectomy and RAI ablation showed soft tissue structures in both thyroid beds bilaterally that could represent residual or recurrent thyroid tissue, although the non stimulated thyroglobulin was very low (0.2 ng/ml) and thyroglobulin antibodies were negative. Table 1 depicts a summary of the six cases.

**Table 1:** Summary of characteristics of six patients with concurrent follicular thyroid carcinoma and papillary thyroid carcinoma.

Case	Age	Sex	Country	Path	Side	Focus	Size (cm)	AJCC stage	Margins	Invasion			EE	Rx	Follow up	
										L	PN	AI			Scan	Lab
1	31	F	Egypt	FTC	L	U	5 × 4	pT3 N0	UI	N	N	N	N	L hemi T; completion R hemi T; 2 fractionated doses RI (30 mci)	No evidence of RI avid local/distant path; No residual/recurrence in TY	Very low TG (<0.1 ng/mL) and TGA (<0.9 IU/mL)
				PTC	L	U	1.3	pT1b N0		N	N	N	N			
2	61	M	Sudan	FTC	R	U	6 × 3 × 2.7	pT3a Nx	UI	N	N	Y	N	R hemi T; completion L hemi T; high dose RI (100 mci)	No evidence of residual TY tissue/metastatic tumor/focal lesion in TY bed	Very low TG (<0.2 ng/mL) and TGA (<0.9 IU/mL)
				PTC	R	U	0.3	pT1a Nx	UI*	—	—	—	—			
3	59	M	Sudan	FTC	L	U	5	pT3a Nx	I	Y	N	N	N	Completion L T; RI ablation 100 mci	Residual TY tissue; received another 30 mci RAI. US Neck follow up showed no residual TY tissue	TG 3.6 ng/mL, TGA 1.2 IU/mL
				PTC	L	U	1.5	pT1bp Nx	I	N	N	N	N			
4	56	F	India	FTC	L	U	4.5 × 3.5 × 2.5	pT3a Nx	I	Y	N	N	N	Total T, then post T high dose RI ablation	Patient travelled out of the country, lost to follow up	
				PTC	L	M	0.6 MD	pT1a Nx	UI*	—	—	—	—			
					R	M	1 × 0.8 × 0.7	pT1a Nx	UI*	N	N	N	N			
					R	M	0.5 MD	pT1a Nx	UI*	—	—	—	—			
5	35	F	Philippines	FTC	R	U	1.3	pT1b N0	UI	N	N	N	N	Total T	Patient travelled out of the country, lost to follow up	
				PTC	R	M	0.8	pT1a N0	UI*	N	N	N	N			
6	52	F	Qatari	FTC	R	U	2.7 × 1.5 × 1.5	pT2 Nx	UI	N	N	N	N	Total T, then post T low dose RI ablation	Residual TY tissue; US Neck follow up showed residual TY tissue	TG 0.2 ng/mL, TGA < 0.9 IU/mL
				PTC	R	U	0.8 × 0.6 × 0.4	pT1a Nx	UI*	N	N	N	N			

* Micro papillary carcinoma; AI Angio-invasion; AJCC American Joint Commission pTNM [9]; EE Extrathyroid extension; F female; FTC; follicular thyroid carcinoma; I involved; L left; L lymphatic; Lab laboratory; M male; M multifocal; MD maximum dimension; N no; Path Pathology; PN Peri-neural; PTC papillary thyroid carcinoma; R right; RI radioactive iodine; Rx treatment; Rx treatment; T thyroidectomy; TG thyroglobulin; TGA thyroglobulin antibodies; TY thyroid; U unifocus; UI uninvolved; Y yes.

## 3 Discussion

PTC and FTC are both derived from thyroid follicular cells, where PTC is the most common and FTC the second most common of all thyroid carcinomas [10]. Both PTC and FTC are differentiated thyroid carcinoma that comprises 90% of all cases of thyroid cancer (incidence about 0.5–10 new cases per 100,000 population globally) [[11], [12], [13]]. Thyroid carcinomas account for about 4% of new cancer cases in the United States [14]. In Qatar, thyroid cancer is the sixth most common cancer across all nationalities and genders; the second most common malignant cancer among females of all nationalities; and the second and fourth most common malignant cancer among non-Qatari and Qatari females respectively [15].

Despite the relatively high prevalence of thyroid cancer, the synchronous co-occurrence of multiple, distinct sub-types of primary thyroid carcinomas is uncommon. In the literature, up to 2017, very few cases of synchronous PTC and FTC have been reported, including three with additional medullary carcinoma, one with additional undifferentiated carcinoma [[3], [4], [5], [6],^16^,^17^], and in one patient, follicular carcinoma, an occult papillary carcinoma and a medullary carcinoma [7].

Table 1 depicts the summary of characteristics of our six patients with concurrent FTC and PTC. In terms of age, three of our patients agree with the age range of the published literature; however, one patient was 31 years old, younger than the mean age. As for invasiveness, among the six cases we observed, the PTC did not exhibit lymphatic, peri-neural or angio-invasiveness; however, the FTC in two out of the six patients (Cases 3 and 4) had lymphatic invasiveness, and in one patient, it showed angioinvasion (Case 2).

In terms of origin, differentiated thyroid carcinomas, e.g., PTC and FTC originate from follicular epithelial cells derived from median endodermal analogues [18]. Whilst the synchronous coexistence of more than one type of thyroid cancer could be a coincidence, several theories have been postulated as potential explanations for such synchronous coexistence. These include propositions that they might be linked to the presence of RET protooncogene mutation in both papillary and medullary thyroid cells [19]; ‘common stem cell theory’ [20]; or common tumorigenic stimulus such as radiation exposure that promotes the malignant transformation of both endodermal and neural crest-derived cell lines [21]. Reports also postulate that the pathogenetic mechanisms of hybrid tumors include collision theory that proposes simultaneous multifocal origin from different cell clones [22], suggesting that two independent tumours are located in the same lesion by simple coincidence [18]; or hostage theory that proposes that adenomatous areas are sequestrated by another tumour type, though the exact etiology is elusive [1]. Where components were separated by normal thyroid tissue, others suggested that the occurrence of e.g., concurrent MTC/PTC is mostly a simple reflection of incidental papillary microcarcinoma, and that concurrent MTC and PTC in the same thyroid should be considered as coincidental [18]. In terms of laterality, five of our cases had both the FTC and PTC in the same lobe. In addition, Case 4 had PTC in both lobes. Likewise, three of our patients (Cases 2, 4 and 5) had an FTC and a micro PTC in the same lobe (PTC measuring from 0.3–1 cm) suggesting that the PTC in these cases was incidental.

As for the pathological assessment, all the patients presented in this case series were examined by the same pathologist at the same laboratory in the same institution, in agreement that cautious and vigilant pathological assessment is critical in disclosing such patterns of pathology. Such vigilance is reflected in our pathology assessment of this case series that involved undertaking many ultra-thin sections that enabled the detection of micro PTC.

As regards to staging and risk prediction, in line with others, we undertook AJCC staging system and ATA stratification prediction system as they are the best predictors of mortality and recurrence respectively [23]. We also employed the AJCC 8th edition that better differentiates differentiated thyroid carcinoma (DTC) risk recurrence for early stages of disease compared with the 7th edition [24].

In terms of treatment, prognosis and survival, we instigated prompt treatment, and four of six patients were cured from the disease, in agreement with that DTC is usually curable when discovered at an early stage [25], and that thyroid malignancies have good long-term prognosis, as early and appropriate treatment yields good results [26]. Likewise, with full preoperative evaluation and stringent follow-up after surgery at our tertiary care institution, for four patients in this series, we observed no recurrence and good survival, in support that the prognosis for DTC is excellent after appropriate surgical treatment, thorough preoperative assessment and strict postoperative follow-up [27]. Two of our patients travelled out of the country and were lost to follow up. In Qatar, the 3-year survival from malignant thyroid cancer during the period 2013–2015 was 90.0% (47.3–98.5%) [15]. Our findings support that the prognosis of differentiated thyroid carcinoma is favorable, with a 10-year survival rate of 80–95% [28], and that earlier detection of small differentiated thyroid cancer with less extensive disease and standardization of treatment may contribute to the decreased disease-specific mortality of such patients [29].

## 4 Conclusions

The patients presented in this case series had different ethnicities, and all had concurrent FTC and PTC in the same thyroid lobe. No apparent cause was found. Four of the six patients were cured, with no recurrence and good survival, whilst the remaining two patients travelled out of the country and were lost to follow up. Endocrinologists and pathologists should be vigilant, aware of and suspicious to the possible simultaneous occurrence of these types. Given the very few cases reported in the literature, further search for the unusual simultaneous occurrence of FTC and PTC is warranted.

## Declaration of Competing Interest

Nothing to declare.

## Sources of funding

Nothing to declare.

## Ethical approval

Approved by medical research center, Hamad Medical Corporation reference number (MRC 17256/17).

## Consent

Written informed consent was obtained from four patients for publication of this case report and accompanying images. A copy of the written consents is available for review by the Editor-in-Chief of this journal on request.

Two patients travelled out of the country after surgery. Written informed consent was not obtained from these patients. The head of our medical team has taken responsibility that exhaustive attempts have been made to contact the family and that the paper has been sufficiently anonymised not to cause harm to the patients or their families. A copy of a signed document stating this is available for review by the Editor-in-Chief of this journal on request.

## Author contribution

**Abdelrahman Abdelaal:** Conceptualization, Data curation, Investigation, Methodology, Project administration, Writing - review & editing. **Walid El Ansari:** Conceptualization, Data curation, Investigation, Methodology, Project administration, Writing - original draft, Writing - review & editing. **Abdelrahman Abusabeib:** Data curation, Writing - review & editing. **Hanan Farghaly:** Data curation, Validation, Writing - review & editing. **Abdelhakem A.M. Tabeb:** Data curation, Writing - review & editing. All authors read and approved the final manuscript.

## Registration of research studies

Not first in Man, hence UIN not required.

## Guarantor

Walid El Ansari: welansari9@gmail.com.

## Provenance and peer review

Not commissioned, externally peer-reviewed
